# Making gene editing accessible in resource limited environments: recommendations to guide a first-time user

**DOI:** 10.3389/fgeed.2024.1464531

**Published:** 2024-09-25

**Authors:** Shivani Goolab, Janine Scholefield

**Affiliations:** ^1^ Bioengineering and Integrated Genomics Group, Future Production Chemicals Cluster, Council for Scientific and Industrial Research, Pretoria, South Africa; ^2^ Department of Human Biology, Faculty of Health Sciences, University of Cape Town, Cape Town, South Africa; ^3^ Division of Human Genetics, Faculty of Health Sciences, University of the Witwatersrand, Johannesburg, South Africa

**Keywords:** CRISPR-Cas9, LMIC (low- and middle-income countries), cost-effective, beginner, genome engineering, genetic diversity, Africa, chromatin landscape

## Abstract

The designer nuclease, CRISPR-Cas9 system has advanced the field of genome engineering owing to its programmability and ease of use. The application of these molecular scissors for genome engineering earned the developing researchers the Nobel prize in Chemistry in the year 2020. At present, the potential of this technology to improve global challenges continues to grow exponentially. CRISPR-Cas9 shows promise in the recent advances made in the Global North such as the FDA-approved gene therapy for the treatment of sickle cell anaemia and β-thalassemia and the gene editing of porcine kidney for xenotransplantation into humans affected by end-stage kidney failure. Limited resources, low government investment with an allocation of 1% of gross domestic production to research and development including a shortage of skilled professionals and lack of knowledge may preclude the use of this revolutionary technology in the Global South where the countries involved have reduced science and technology budgets. Focusing on the practical application of genome engineering, successful genetic manipulation is not easily accomplishable and is influenced by the chromatin landscape of the target locus, guide RNA selection, the experimental design including the profiling of the gene edited cells, which impacts the overall outcome achieved. Our assessment primarily delves into economical approaches of performing efficient genome engineering to support the first-time user restricted by limited resources with the aim of democratizing the use of the technology across low- and middle-income countries. Here we provide a comprehensive overview on existing experimental techniques, the significance for target locus analysis and current pitfalls such as the underrepresentation of global genetic diversity. Several perspectives of genome engineering approaches are outlined, which can be adopted in a resource limited setting to enable a higher success rate of genome editing-based innovations in low- and middle-income countries.

## 1 Introduction

Clustered regularly interspaced short palindromic repeat sequences (CRISPR), was first discovered as an uncharacteristic prokaryotic DNA repeat element then later identified as the bacterial adaptive immune system and subsequently harnessed or repurposed as a versatile reprogrammable gene-targeting platform (Ishino et al., 1987; Jansen et al., 2002; Adli, 2018). The versatility of CRISPR-based genome editing has enabled a myriad of genetic perturbations in eukaryotic cells (Cong et al., 2013; Jinek et al., 2013; Mali et al., 2013). This includes insertions and deletions (indels) to disrupt gene function, precise base alterations and fragment deletion or insertion to restore gene function (Sander and Joung, 2014). The Cas9-gRNA complex inspects the genome for protospacer adjacent motif (PAM) sequences within the targeted site, inducing conformational changes of Cas9 to mediate a double stranded break (DSB) by the nuclease domains of Cas9 (Jinek et al., 2013; Sternberg et al., 2014). It is the double stranded break (DSB) that prompts the primary (commonly known) endogenous repair pathways, non-homologous end joining (NHEJ, Box 1), microhomology mediated end joining (MMEJ, Box 1) and homology directed repair (HDR, Box 1), exploited by CRISPR-Cas9 in single guide RNA (gRNA)-dependent manner to mediate these perturbations (Jeggo, 1998; Jinek et al., 2012; Cho et al., 2013; Mali et al., 2013; Hsu et al., 2014; Sander and Joung, 2014; Graham and Root, 2015). Compared to older generation endonucleases such as TALENs, which require challenging protein engineering at 3-6 fold greater cost per reaction (*Gene-Editing Could Modify and Cure Disease: CRISPR* vs. TALENs, 2017), the sequence specific base-pairing nature of the CRISPR gRNA lends itself to be more flexible, simpler to use and multiplexable (Cong et al., 2013; Jinek et al., 2013; Cox et al., 2015).

**Box 1 genome engineering terminology**

HDR an endogenous, less efficient (activity is cell cycle restricted) repair pathway mediated after DNA damage, DSB formation, in the presence of a homologous repair template, thereby permit precise modifications to the target sequence.

NHEJ an endogenous, more efficient (activity is unrestricted throughout the cell cycle) yet error-prone repair performed after DSB formation, in the absence of a homologous repair template that involves the ligation of DNA ends perfectly or with indels.

MMEJ an endogenous, less efficient (activity is cell cycle restricted) alternative to NHEJ repair that involves the ligation of DNA between identical micro homologous sequences flanking the DSB site.

NMD is considered a translation dependent surveillance system, which degrades aberrant transcripts containing PTC to prevent the synthesis of truncated proteins.

Internal ribosome entry sites are secondary mRNA structures, which recruit the ribosomal subunit to prompt translation initiation. These elements can be inserted between multiple genes allowing for the co-expression of multiple genes from a single mRNA transcript.

Self-cleaving peptides (18–20 amino acids in length) produce equivalent ratios of multiple genes from the same mRNA by ribosome skipping.

Serine recombinases create genetic modifications as specific DNA sites and recombination crossovers can occur between attachment, attP (acceptor) sites which are landing pads, a prerequisite for integration at the target locus and the attB (donor) sites residing in DNA cargo cassette to be inserted.

Cre-Lox recombination mediates the rearrangement of DNA by inversion, excision and translocations, whereby Cre recombinase recognizes specifically located and orientated loxP sites on DNA.

CRISPR-Cas9 genome engineering technology can address global health challenges, for example, efforts aimed at eradicating malaria and challenging the burden of HIV, which is realized in the Global North. Barriers to adopting genome engineering studies in the Global South include the low prioritization of resources and research budgets for science and technology, the lack of accessibility and affordability for these reagents including infrastructure generated in the Global North, and the dearth of expertise in utilizing this tool (UNESCO Institute for Statistics; OECD Main S&T Indicators; DSI/HSRC 2019/20 R&D Survey Report). In a 2022 report on accelerating the access to genomics for global health the WHO argued, “It is not justifiable ethically or scientifically for less-resourced countries to gain access to such (genomic) technologies long after rich countries do,” and this statement holds truth in the field of gene editing (WHO, 2022). An analysis of literature published globally on the topic of “CRISPR gene editing” technology was summarized by Abkallo and colleagues (2024). It is evident from these findings that high-income Countries have the highest impact with a wide network of existing collaborations, which is lacking but proliferating in low- and middle-income countries (LMIC) and upper middle-income countries (Gao et al., 2021). It is imperative for LMICs to gain the same benefits acquired with the utility of this genome engineering platform. This could be solved by collaborative efforts both nationally and internationally, providing training, more private and governmental funding and alignment with existing genome engineering strategies used by the Global North to create a more cost-effective approach. Whilst these key recommendations to overcome the challenges for genome editing-based innovations are discussed elsewhere, including detailed regulatory and ethical issues (Caelers, 2023; Abkallo et al., 2024), the purpose of this review is to highlight theoretical and practical considerations aimed at reducing the cost of performing efficient and precise genome engineering *in vitro*, which can be easily implemented by a first-time user based in any molecular biology laboratory with limited resources. The considerations provided in this review are not aimed towards improving the cost effectiveness of direct therapeutic applications using CRISPR-Cas9 genome editing, as the financial resources presiding over treatment development for gene therapy remains challenging even for the Global North. Here, we discuss the necessity of democratizing this genome engineering technology for LMICs as a molecular tool for researchers limited by resources. In addition, we take inventory of CRISPR-Cas9 based mutagenesis studies to provide cost effective strategies that keep pace with the required efficacy and precision if this cutting-edge technology is to realize its potential within a resource limited setting.

## 2 Understanding and dissecting the target locus

### 2.1 The need for genetic diversity to empower bioinformatic tools for representative research

The underrepresentation of genomic data from the African continent has biased global studies, for example, by extrapolating genetic risk from studied, European populations less relevance has been provided to this population (Sirugo et al., 2019). The ‘homing mechanism’ of Cas endonucleases is coordinated by the gRNA, making it a core component that is easily optimised by *in silico* algorithms at no cost. Existing computational algorithms for the design of these CRISPR components including the gRNA, repair template and primers make use of existing reference genome sequences (e.g., GRCh38), which primarily represent the European population and lack genetic diversity. This lack of representation within these existing databases biases the outcome of the gene edit generated in African cell lines, which are genetically diverse and harbour unique or highly prevalent variants that are absent in reference genomes (Canver et al., 2018; One pangenome to bind them all, 2022; Misek et al., 2024). Reduced experimental efficiency and greater cost is incurred when inaccurate gRNAs are designed against a less inclusive reference genome sequence. This results in low, or absent cleavage at the target locus and/or cleavage at loci of high sequence similarity referred to as off-target sites, which may lead to negative confounding effects and therefore would require additional design and experimental analysis (Cancellieri et al., 2023).

Mismatches in the gRNA sequence proximal to the PAM restrict Cas9 endonuclease cleavage by greater than 2000-fold, achieving 20% cleavage activity in comparison to a gRNA sequence without such mismatches to the target locus (Bravo et al., 2022). As an example, a gRNA was predicted to mediate high editing efficiency, using the existing GRCh38 sequence, at the target locus, *CYP3A5* in cells of African origin. The variant, rs4646450 G>A located within *CYP3A5* exists in 97% of the African population compared to 17% prevalence in the European population. However, this variant resides proximal to the PAM of a predicted gRNA and may potentially ablate Cas9 cleavage in the cells of African origin. This disadvantages studies involving targeted regions with unknown/or rare variants. For this reason, it is imperative when selecting optimally active CRISPR components for genome engineering studies that the reference sequence used accurately represents this genetic variation to achieve the required outcome (Kwart et al., 2017; Canver et al., 2018).

Several publications have demonstrated the importance of including genetic variation in genome editing design strategies (Lessard et al., 2017; Cancellieri et al., 2023; Li et al., 2023; Misek et al., 2024). Users can find relevant variant information from accessible public databases such as gnoMAD. In addition, whilst there is a dearth in extensive computational expertise required to manipulate large variant datasets, computational tools for gRNA design such as CRISPOR provides support for researchers to import alternative sequencing datasets (by direct correspondence) (Haeussler et al., 2016; Concordet and Haeussler, 2018). Furthermore, a recently developed computational tool, CRISPRme, predicts gRNA off-target sites by integrating human genetic variant datasets (Cancellieri et al., 2023). However, as a simple solution, one would always recommend sequencing the region of interest from the cell type of intended modification, which provides a baseline sequence with which to design accurate and population relevant gRNAs.

### 2.2 Chromatin landscape impacts gene editing efficiency and the balance of repair pathways

Achieving enhanced editing efficiency comes at increased reagent costs and time required to perform research (*CRISPR Benchmark survey* HubSpot, 2020). However, this can reduce time associated with the successful isolation of an edited clone. This is most easily measured by assessing the level of edits in a global population of cells prior to clonal expansion. If the global population of editing is low (<10%), hundreds of clones must be screened to identify multiple isogenic clones.

**FIGURE 1:**
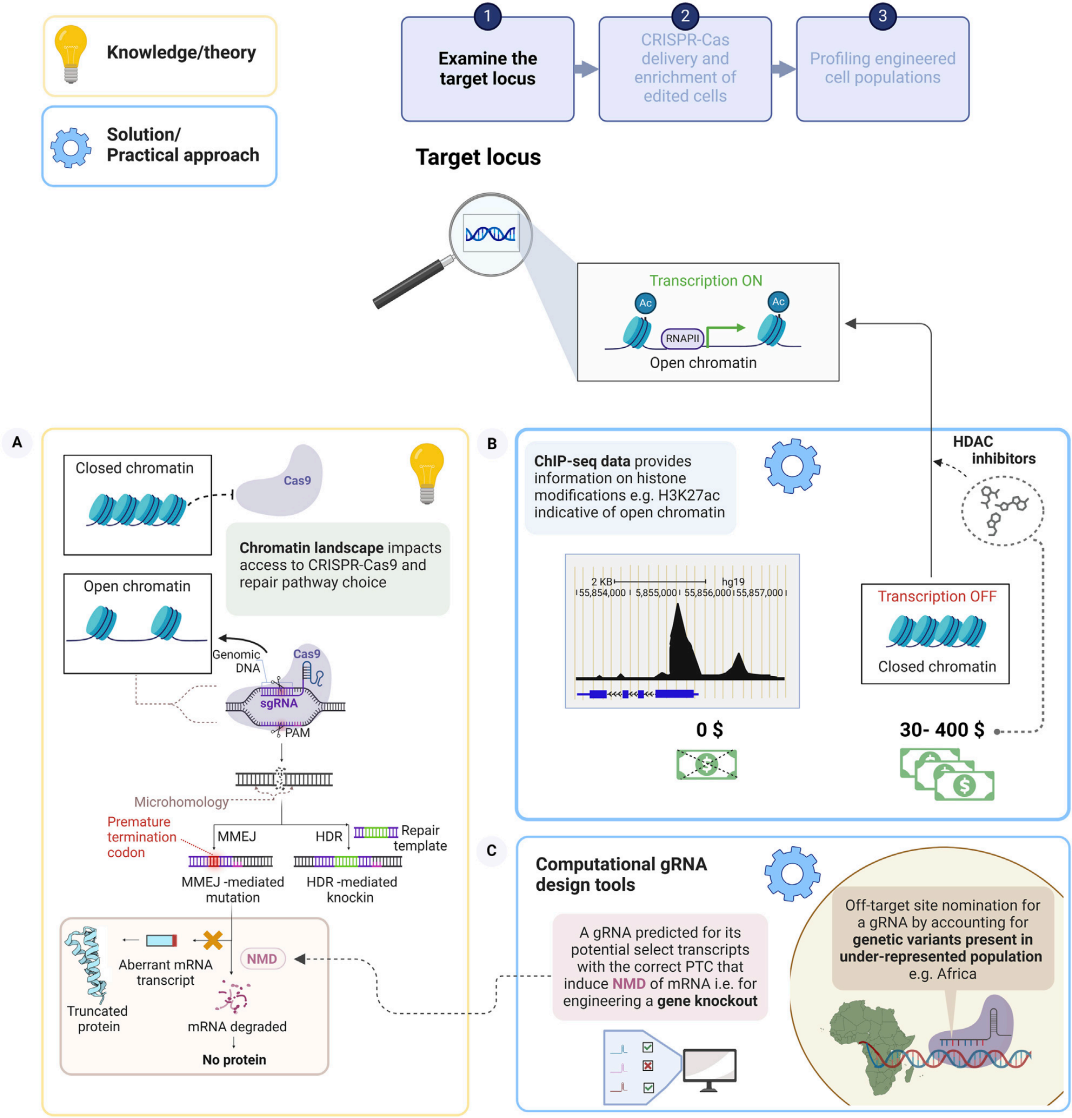
The chromatin environment of the target locus impacts the performance of CRISPR-Cas9 to influence the outcome of the gene edit. **(A)** Schematic of CRISPR-Cas9 mediated DSB cleavage of a target locus with accessible chromatin, under gRNA guidance. This elicits the endogenous repair pathways, whereby a homologous sequence (sister chromatid), repair template could be precisely inserted via HDR. Alternatively, in the absence of a repair template, deletions flanking microhomologous sequences in proximity to the DSB are mediated by MMEJ, and example of error-prone repair, which may disrupt the coding region and incorporate a PTC. Aberrant transcripts escape the mRNA surveillance pathway, Nonsense-mediated decay (NMD) to form truncated protein, whereas transcripts that meet the criteria for NMD are degraded leading to a gene knockout. **(B)** Chromatin immunoprecipitation sequencing data sets, for example, of open chromatin associated histone marks at the target locus could be examined, at no cost to the user to ensure optimal gRNA efficiency. Epigenetic drugs may improve CRISPR-Cas9 editing efficiency by chromatin decompaction **(C)** Examples of computational gRNA design tool to nominate gRNAs, which will ensure gene knockout and to evaluate on-target and off-target sites by including genetically diverse variants in the design. Created with BioRender.com.

Therefore, understanding and dissecting the chromatin landscape of the target locus is crucial to improving gene editing efficiencies, as it dictates the “searching and binding” function of Cas9-gRNA to the target site shown in Figure 1 (Wu et al., 2014; Chari et al., 2015). Heterochromatin regions consist of tightly packed DNA, which generally occludes access of Cas9 to the target DNA, due to the constrained accessibility of the DNA to Cas9 binding and cleavage, (left schematic, Figure 1) (Kallimasioti-Pazi et al., 2018; Klemm et al., 2019; Schep et al., 2021). Consequently, the outcome of CRISPR-Cas9 gene editing efficiencies is dependent on the accessibility of the chromatin at the specific loci targeted (Verhagen et al., 2022; Schep et al., 2024). Accumulating evidence has shown the relative abundance of indels generated by MMEJ (Figure 1, right schematic) or NHEJ (McVey and Lee, 2008), with larger indel sizes attributed to MMEJ, is modulated by the chromatin landscape, which influences the indel profile of the targeted site (Chakrabarti et al., 2019). These profiles are dictated by differences in both histone acetylation levels and cell types (Schep et al., 2021; 2024). This can be mitigated by decompacting heterochromatin at targeted loci with epigenetic drugs (histone deacetylase inhibitors).

Chromatin remodelling compounds such as tubastatin A and trichostain A have been employed to enhance editing efficiency where chromatin state may be refractive to editing. HDAC inhibitors regulate various cellular pathways; therefore, careful evaluation is required in the use of these compounds. Tubastatin A regulates several cellular processes, including activation of cell cycle arrest and is considered a therapeutic for several diseases, such as cancer, ischaemic stroke and Alzheimer’s disease (Falkenberg and Johnstone, 2014; Fan et al., 2018; Ling et al., 2020; Shen et al., 2021). *In vitro* models implicate this HDAC inhibitor as a regulator of neurogenesis, altering the dominance of emerging neuronal and glial cell types during differentiation (Iaconelli et al., 2017). Furthermore, it was shown that the developmental potential of mouse embryos was negatively impacted by tubastatin A treatment (Wang et al., 2019). Exposure of trichostatin A to cells induces activation of ataxia-telangiectasia mutated kinase, which acts on various DNA damage responses, including induction of cell cycle checkpoints, DNA repair, and apoptosis (Maya et al., 2001; Bakkenist and Kastan, 2003; Lee, 2007). Although this compound activates global histone acetylation and DNA methylation effects, its function represents both locus specificity and site selectivity. Additionally, this inhibitor indirectly triggers demethylation in nondividing cells, a function considered specific to DNA methyltransferases inhibitors (Ou et al., 2007).

Tubastatin A and trichostain A cost $218 and $652 for 5 mg, respectively, these compounds may be beyond the financial limits of researchers based in LMICs. However, the compounds are generally used at low concentration (in the nM–uM, range) during chromatin remodelling studies and can be stored long term; therefore, this may be a prudent long-term investment. Tubastatin A was shown to increase editing efficiency by 1.2-fold at 15 loci in retinal pigment epithelial cells (Schep et al., 2024), while trichostatin A supported Cas9 editing in HepG2s led to a 2-fold increase at four loci with repressive chromatin environments but was not as effective at two other loci that have higher H3K27ac levels (Chakrabarti et al., 2019). Consequently, a decision to use chromatin modulators should balance a) cost, b) efficacy at different target loci and cell types, with c) potential detrimental downstream effects on global transcription (Riesenberg and Maricic, 2018; Sandonà et al., 2023). At present, a cost-effective approach would be to evaluate the maps of euchromatin or heterochromatin features from available genome wide Chromatin immunoprecipitation sequencing (ChIP-seq) (Johnson et al., 2007) data, providing a genome-scale map of DNA-protein interactions such as nucleosome positioning, histone modifications, DNA methylation and transcription factors. Profiling DNA-protein interactions such as the H3K27ac, histone modification can significantly improve the selection of an “optimal” target loci for CRISPR-Cas9 gene editing, (Figure 1, right schematic). Not all gRNA selection algorithms consider the effect of the chromatin landscape on CRISPR-Cas9 performance and gRNA binding, further restricting a first-time user. Yet, by allocating sufficient time to the design aspect of the gene edit and incorporating an assessment of the chromatin landscape to ensure the target loci displays open chromatin status (Zhang et al., 2021), these pitfalls can be navigated. These strategies underscore the importance of pre-empting high costs and reduced efficiencies by meticulous examination of the target loci. This involves dissecting the chromatin status of the target, accounting for diverse genetic variants and negating potential negative consequences, which may arise post genome editing, thereby impacting the design of CRISPR components.

### 2.3 Examples of harnessing bioinformatic gRNA design tools for efficient editing

#### 2.3.1 Widely used gRNA design tools

Existing gRNA selection algorithms factor Cas9 binding and hence cleavage of closely matched target loci, high gene editing efficiencies with reduced off-target effects (Fu et al., 2013; Hsu et al., 2013; Doench et al., 2016). The target loci are screened to identify PAM sites in the vicinity of the desired edited region. CRISPOR provides scores on the specificity, efficiency, knockout and off-target sites of several gRNA sequences based on several PAM sequences at the target locus provided by the user. Additionally, primers sequences for cloning the gRNA into widely used plasmids are provided, along with primer sequences for clone screening and off-target sites analysis, post gene modification (Haeussler et al., 2016; Concordet and Haeussler, 2018). An alternate computational gRNA design tool, CHOPCHOP, allows for the gene modification, such as gene knockout and knockin as an input. Similarly, gRNA scores, homology arm design for the repair template to generate gene knockin and off-target sites are provided (Montague et al., 2014; Labun et al., 2019). Comprehensive overviews of other available gRNA design tools for gene editing have been reviewed elsewhere (Liu et al., 2019; Alipanahi et al., 2023).

#### 2.3.2 gRNA design to achieve gene knockout

In the absence of a precise repair template, the primary repair mechanism, following a DSB, is error prone repair (e.g., MMEJ/NHEJ), which is frequently exploited to create indels, leading to frameshift inducing premature termination codons (PTCs). These can potentially elicit nonsense-mediated decay (NMD, Box 1) (Lykke-Andersen et al., 2000; Brogna and Wen, 2009), a common strategy employed to generate gene knockouts, Figure 1 (left schematic). However, Tuladhar and colleagues (2019) showed that indels can induce internal ribosome entry sites (IRES, Box 1), (Terenin et al., 2017). This differential translation caused by the presence of indels produces alternative mRNA transcripts or induce exon skipping producing aberrant, truncated proteins which may escape NMD to exert a dominant negative function (Green et al., 2003). This study developed a free-to-use computational tool for gRNA selection to avoid these events by providing a prediction of exon skipping at on-target and off-target sites to select for PTC that elicit NMD for generating a true gene knockout as shown in Figure 1 (right schematic) (Tuladhar et al., 2019). The computational tool has been successfully used in combination with the conventional gRNA design algorithms at MIT, www.crispr.mit.edu (Ran, et al., 2013b) and the CHOP-CHOP algorithm (http://chopchop.cbu.uib.no/) (Montague et al., 2014) to select for optimal gRNA when creating gene knockouts (Burckhardt et al., 2021; Seumen et al., 2021).

#### 2.3.3 gRNA design for MMEJ-based gene modifications

The MMEJ repair pathway is typically of use as an alternative method to HDR, to mediate gene knockin termed Precise Integration into Target Chromosome (PiTCH) system. Multiple DSBs are induced to allow microhomologies to anneal and incorporate the donor vector. Detailed vector cloning and design strategies including donor vector construction, are described to perform these modifications (Sakuma et al., 2016). Furthermore, this repair pathway is commonly used to generate relevant deletion mutations using paired gRNAs. The joining of microhomologous sequences flanking DSBs is exploited to generate predictable and precisely sized deletions (McVey and Lee, 2008). Deletion mutations account for 25% of genetic variation, which may be flanked by microhomologous sequences, making them amenable to MMEJ based editing. The gRNA design tool, MHcut predicts targetable microhomologies, which are used to create biologically relevant deletion mutations for disease modelling and aid in drug screening platform development (Grajcarek et al., 2019). Similarly, MMEJ-KO allows for the design of paired gRNAs to take advantage of MMEJ-based genomic deletions (Xie et al., 2021).

#### 2.3.4 Considerations for repair template design

The HDR repair mechanism mediates precise modifications, such as incorporating a SNP. To enhance HDR efficiency, the DSB generated by CRISPR-Cas9 must occur within 10 bp from the modification to achieve homozygous mutations and within 5–25 bp from the DSB site to create heterozygous mutations (Paquet et al., 2016). The number of correctly edited HDR reads generated after targeting the PSEN1 locus, increased from 40%, when the DSB was mediated 20 bp from the mutation, to >90% when this distance (DSB to mutation) occurred within 5bp, in iPSCs. A comparable improvement in HDR efficiency was observed at the same locus in HEK293Ts, suggesting a general DSB to mutation distance governs HDR efficiencies in these cells. Furthermore, to prevent CRISPR-Cas9 from recutting the modified site and generating NHEJ-mediated indels, silent mutations are introduced in the designed repair template to mutate the PAM recognition sequence or gRNA binding sequence. For example, the PAM recognition site for SpCas9 can be altered from AGG to CGA, as both code for arginine (Paquet et al., 2016; Kwart et al., 2017). PAM blocking mutations improved HDR efficiencies at the PSEN1 locus in HEK239T and iPSCs by 4-fold and 2-fold, respectively, which suggests a dependency on cell type. This improvement in the efficiencies increases the probability of obtaining accurately modified cells, reducing the cost of reagents required for both cell culture and validation of the gene modification. Care must be taken in repair template design as the non-canonical PAM sequences (NAG and NGA) can be recognized by SpCas9 though less effectively, and must thus be avoided (Hsu et al., 2013). In addition, the incorporation of silent mutations within the repair template, which introduce restriction enzyme recognition sites can expedite the selection of precisely edited clones (Botstein et al., 1980; Chen et al., 2011; Jinek et al., 2012; Ran, et al., 2013a, 2013b). CRISPRcruncher is an algorithm that considers codon degeneracy to identify silent restriction enzyme recognition sites, which are ideally introduced in proximity of the precise mutation (Fay et al., 2021). CRISPR Knock-in Designer is another web-based tool that requires information on the target site of interest, gRNA sequence and gene ID as the input, to create a repair template with optimized silent mutations for the target locus. In addition, this computational tool provides primer sequences for the screening of clones (Prykhozhij et al., 2021). These strategies described for repair template design can be implemented at no cost and will save significant experimental time and resources.

#### 2.3.5 Off-target mitigation strategies by altering gRNA sequence length and composition

Higher-fidelity Cas9 variants have been engineered to reduce off-target effects, but this strategy does not completely eliminate the unintended effects and furthermore result in reduced on-target cleavage activity (Kleinstiver et al., 2016; Slaymaker et al., 2016; Chen et al., 2017; Vakulskas et al., 2018). A cost-effective alternative to reducing off-target effects and cytotoxicity caused by DSBs involves the direct modification of the gRNA. GUARD RNAs are 14–16 bp with an intact PAM sequence. The GUARD RNA sequence is positioned 12–25 bp downstream of the conventional gRNA sequence, which binds with complete homology to off-target loci, thus forming catalytically inactive complexes with Cas9 rendering these genomic regions inaccessible for cleavage (Dagdas et al., 2017; Coelho et al., 2020). CRISPR Guide RNA Assisted Reduction of Damage (GUARD) Finder was developed from this study as a free access online tool to increase specificity at targeted loci (Coelho et al., 2020). Despite the ease in implementation, this strategy poses drawbacks such as optimizing the molar ratios of the conventional gRNA and GUARD RNA added to target cells to ensure off-target binding by the GUARD RNA. Another tunable system aimed at restraining CRISPR-Cas9 activity was created by the insertion of cytosine stretches to the 5′-end of the gRNA sequence to decrease off-target effects. This safeguard-gRNA strategy reduced p53-mediated cytotoxicity in iPSCs. The addition of shorter cytosine stretches is required to increase biallelic modifications and longer stretches enhanced monoallelic editing at the Cdh1 and VEGFA1 loci in mouse hepatoblast cells and human adipose derived stem cells, respectively (Kawamata et al., 2023). The strategies can be implemented to improve the safety of gene editing but reduce on-target efficiencies.

#### 2.3.6 Off-target analysis by integrating genetic variation

The lack of equitable genetic representation and the negative implications thereof on the efficiency of CRISPR-Cas9 technology on has been discussed above (Canver et al., 2018; Misek et al., 2024). Researchers are making concerted efforts to address this barrier, for example, with the development of computational tool, CRISPRme, which (Figure 1, right schematic) predicts potential off-target sites by accounting for population-level genetic variants (Cancellieri et al., 2023). Predesigned gRNA sequences, using CRISPOR (as an example) are required as an input. This software was used to predict potential off-target sites for the gRNA mediating the disruption of an enhancer, which is a regulatory element that substantially activates gene expression by establishing long interactions with the promoter (Banerji et al., 1981). Targeting this B-cell lymphoma/leukemia 11A (*BCL11A*) enhancer, reactivates fetal hemoglobin expression and is a therapeutic strategy for patients with sickle cell disease. The candidate off-target site, with 3 mismatches to this gRNA sequence, is produced by a SNP common in one of twenty people of African ancestry and introduces a PAM recognition site (Cancellieri et al., 2023). Prior preclinical evaluation, using the reference genome, lacking in genetic diversity, showed no off-target effect of the gRNA (Frangoul et al., 2021). However, a 4% indel efficiency was generated at the homologous gene in hematopoietic stem cells of a donor heterozygous for the SNP. It should be noted that the data generated using CRISPRme are not transferred or stored online, thus respecting genomic privacy and settings and variants can be reported by the user (Cancellieri et al., 2023). Despite efforts towards optimising *in silico* gRNA design, several predicted gRNAs yield low or absent activity at the targeted locus with a dependency on cell type, thus reiterating the importance of experimental evaluation (Xu et al., 2015; Haeussler et al., 2016; Chakrabarti et al., 2019). The use of the described, next-generation software tools (Tuladhar et al., 2019; Cancellieri et al., 2023) illustrates the importance of target locus examination during preliminary design strategies to scrutinize the target locus to achieve successful gene manipulation.

## 3 Consideration for CRISPR-Cas9 delivery format

### 3.1 The cost implications and caveats of CRISPR-Cas9 delivery modalities for *in vitro* application

Transfection is a conventional method employed to deliver molecular cargo into eukaryotic cells. Cas9-gRNA components can be transfected using DNA (plasmid), mRNA or ribonucleoprotein (RNP) formats. It should be noted that the packaging of genes into vectors, *in vitro* transcription and protein translation is not specific to CRISPR-Cas9 delivery. However, the selection of the delivery format may be dictated by the availability of resources allocated to perform genome engineering. Table 1 guides a first-time user (attempting to reduce reagent use and equipment costs) on selecting a CRISPR-Cas9 gRNA delivery format that is centred on the cost effectiveness, transfection efficiency, constraints, and advantages.

**TABLE 1:** Selecting a format for CRISPR-Cas9-gRNA delivery.

SpCas9	Plasmid (excluding viral vectors)	mRNA	RNP	Companies and References
Cost	Low, varying from $150-$ 350 with unlimited use of purchased plasmid	Moderate, varying from $400-$1000 with limited use of purchased reagents	High, varying from $500-$1400 with limited use of purchased reagents	Sigma-Aldrich Addgene ThermoFischer Scientific Integrated DNA technologies
Efficiency	Prone to off-target effects (long term Cas expression)	Low- moderate off-target effects (long term Cas expression)	Minimal off-target effects (short term Cas expression)	(Kim et al., 2014; Liang et al., 2015; Zuris et al., 2015)
	Stable	Poor stability, prone to degradation	Prone to degradation	(Kim et al., 2014; Liang et al., 2015)
	Prolonged activity	Transient intermediate activity	Transient, rapid activity	(Kim et al., 2014; Liang et al., 2015)
	Easily multiplexed	Multiplexable	Multiplexable	(Cong et al., 2013; Esvelt et al., 2013; Kurata et al., 2018; McCarty et al., 2020; Verhagen et al., 2022)
	Labor-molecular cloning (transfection with low endotoxin purified plasmid DNA)	No labor for production (commercial products)	No labor for production (commercial products)	(Butash et al., 2000; *Addgene: CRISPR References and Information*, 2023)
	Cytotoxic (foreign DNA)	Low cytotoxicity	Low cytotoxicity	(Sun et al., 2013; Kim et al., 2014; Hendel et al., 2015)

Approximate cost of reagents in July 2024 with a dollar to ZAR, exchange rate of 18.19.

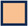
 Disadvantageous 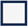 Average 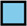 Advantageous.

**FIGURE 2:**
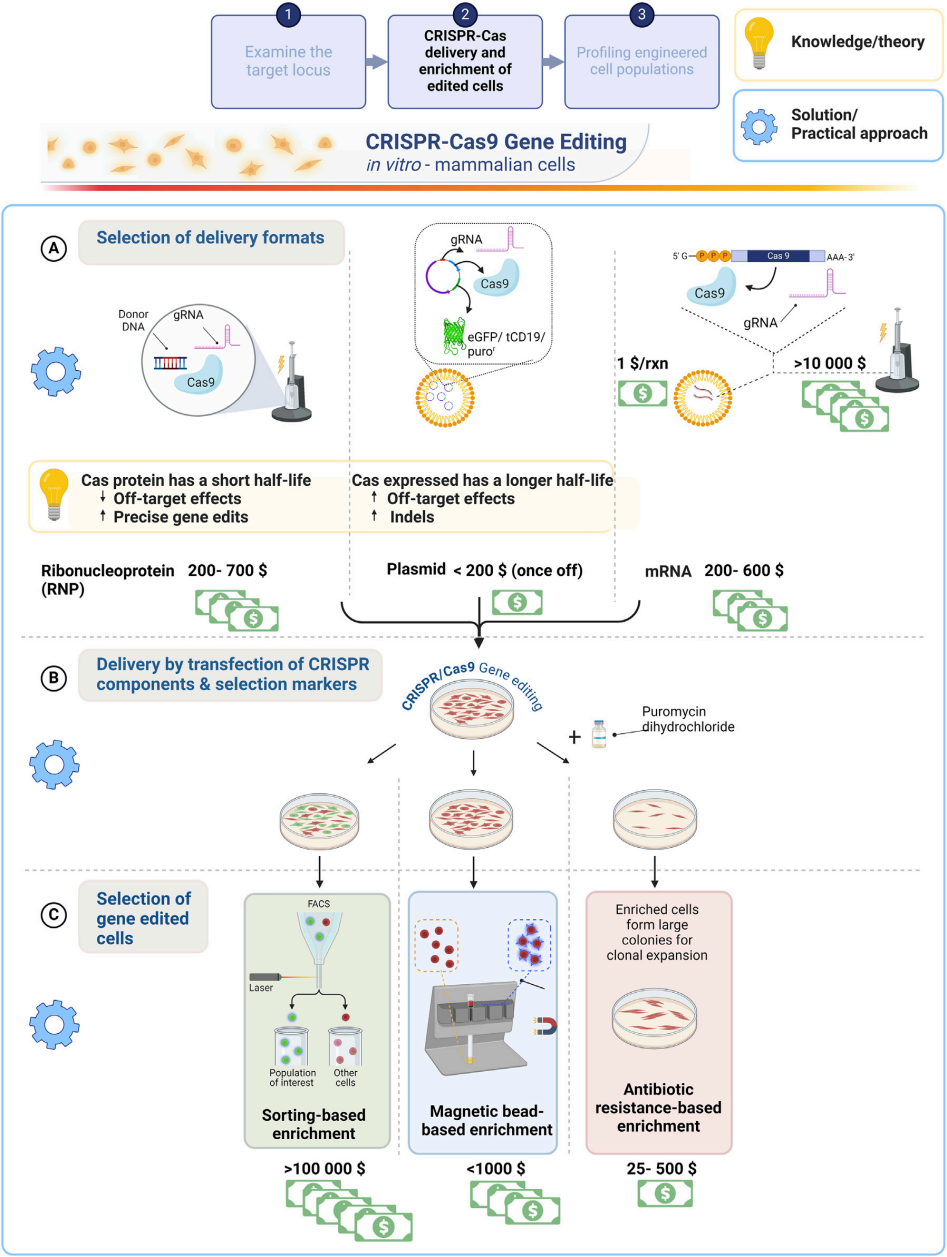
A comparative overview of common CRISPR-Cas9 delivery formats for mammalian cell transfection and enrichment strategies aimed at improving gene editing efficiency with a cost comparison of these strategies. **(A)** Schematic of electroporation and lipid-based import of CRISPR-Cas9 delivered as RNP, plasmid DNA or mRNA into the cell. The cost effectiveness of these methods is compared to assist a first-time user with constrained resources. Of importance is the half-life of Cas9, which will impact the gene editing outcome (yellow box). **(B)** A GFP (FACS), surface marker (MAGECS) and/or antibiotic resistance gene could be used to facilitate the selection of gene edited cells. **(C)** The cost effectiveness including less laborious factors the choice of enrichment strategy selected to enhance efficiencies of obtaining correctly edited clones. Created with BioRender.com.

RNPs are commonly used since transcription and translation is not required, and Cas protein can be administered with the gRNA directly into the nucleus by nucleofection. Furthermore, reduced off-target effects are incurred owing to the shorter lifetime of Cas protein to accomplish “hit and run” gene editing (Kim et al., 2014; Liang et al., 2015). However, the drawback of RNP delivery is the high cost to purchase a single synthetic gRNA and Cas protein or the labour and resources to purify Cas protein in-house. Cas9 mRNA is synthesized by *in vitro* transcription and is delivered to the cytoplasm (efficiently by electroporation) for translation (RNP formation) and nuclear uptake (Glass et al., 2018). These formats (mRNA and ribonucleoprotein) incur low to moderate off-target effects (Yang et al., 2013a; Kim et al., 2014; Liang et al., 2015; Zuris et al., 2015) but are generally more costly (Figure 2A). Cost-effective CRISPR-Cas9 delivery, including theoretical and practical solutions are provided in Figure 2.

Dual expression plasmids encoding Cas9 and gRNA are the most widely used since this was the first format introduced for mammalian gene editing (Ran, et al., 2013b). This method is stable, easily constructed through simple, well established cloning protocols making it the affordable alternative to produce on a low budget compared to the alternatives, specifically when multiple genes are targeted. However, developing the plasmid cloning strategy, primer design followed by the cloning procedure, bacteria transformation, plasmid purification and sequencing requires sufficient time and resources. Commercially available bacteria competent cells cost approximately $10 per reaction, as an cost-effective strategy, chemically- and electro-competent cells can be generated in-house using existing protocols (Sambrook and Green, 2012; Green and Sambrook, 2021). Low-funded research but resourceful labs have minimized costs associated with cloning plasmids by developing homemade plasmid miniprep solutions, including in-house RNase A overexpressed from a bacterial system, which are the core components generally supplied in commercial silica based columns kits. This homemade kit yielded comparable purity compared to a commercial kit. Additionally, the molecular marker (DNA ladder) for performing gel electrophoresis was developed by the amplification of the purified plasmid DNA using seven primer pairs producing PCR products ranging from 100 to 1500 bp (Elnagar et al., 2022). These implementable strategies can release financial resources for essential reagents that are more expensive.

The RNP and mRNA formats supplied in quantities of 10 nmol synthetic gRNA combined with either 250 µg of Cas 9 protein or 25 µg Cas9 mRNA costs approximately twice as much compared to dual expression plasmid DNA. Every locus targeted requires the synthesis of a custom gRNA and therefore, in our experience it is more costly to purchase synthetic gRNAs coupled with the RNP and mRNA formats compared to annealing gRNAs purchased from an oligo synthesis provider. However, the former will reduce the time required for plasmid cloning and purification to ensure high quantity and quality (presence of low endotoxin) Cas9-gRNA expressing plasmids are produced, as described in Table 1 and Figure 2. Higher Cas9 longevity is achieved using the plasmid format compared to RNP, which limits Cas9 exposure (Figure 2A, yellow box). This poses a caveat for the first-time user selecting an affordable delivery format, as the delivery format impacts the outcome and efficiency of precise gene edits (Richardson et al., 2016; Kosicki et al., 2017; Brinkman et al., 2018a). Consequently, the disadvantage of the most cost-effective plasmid format is an increased risk of off-target effects, as expression is detected 72 h post transfections, which prolongs Cas9 activity at the DSB site (Kim et al., 2014; Hendel et al., 2015; Bloomer et al., 2022). This increases the adverse off-target effects, deleterious on-target genomic deletions, p53 mediated cytotoxicity and cell cycle arrest (Merkle et al., 2017; Haapaniemi et al., 2018; Ihry et al., 2018; Kosicki et al., 2018). Several studies have thus focused on regulating Cas9 activity for safer editing, yet the on-target efficiency is generally compromised (Kawamata et al., 2023).

Reduced gene editing efficiencies obtained in a cell population can be mitigated by enriching for cells that have incorporated the desired edit by co-expression of fluorescent tags (e.g., GFP) or antibiotic (e.g., puromycin) resistance genes. This indirectly improves the efficiency by increasing the frequency of selecting an edited cell whilst significantly reducing the labour cost and time involved for clonal expansion (Ran, et al., 2013b; Steyer et al., 2018). While chemical transfection (including lipofection and calcium phosphate transfection) and electroporation are standard methods employed for the delivery of components into a cell, in a resource limited environment the cost is restrictive (Figure 2A). Calcium phosphate co-precipitation mediates endocytic uptake of DNA, which is inexpensive, requiring only calcium chloride solution and HEPES-buffered saline supplemented with sodium phosphate. This method is simple to perform, requires no specialized equipment and the chemical components are easily resourced (Karra and Dahm, 2010; Kwon and Firestein, 2013). Lipid based transfection is less cytotoxic owing to the endocytic uptake of Cas-gRNA complex making it more economical with an approximate cost of $0.5 per transfection performed, but a drawback of this method is reduced editing efficiencies, influenced by cell type incompatibility (Cong et al., 2013; Zuris et al., 2015; Wei et al., 2020). Electroporator permit efficient transfection of cells (HSCs, T-cells), which are less amenable to transfection (Kim et al., 2014; Stadtmauer et al., 2020; Verhagen et al., 2022). However, the disadvantages include high post-transfection mortality and higher expenses varying from $13 000 to $30 000 for equipment, consumables and reagents (Yip, 2020), a challenging feat for a research group with a constrained research budget. Electroporation buffers developed and optimized in-house and the ability to regenerate electroporation tips, provide cost-effective strategies to allow the adoption of this equipment for researchers in LMICs (Chicaybam et al., 2013; Brees and Fransen, 2014). This has been shown through the electroporation of Cas9-gRNA plasmid in peripheral blood mononuclear cells and HEK293FT cells using an in-house developed glucose-based buffer mediated the disruption of the gene encoding inhibitory receptor PD-1 (Chicaybam et al., 2017). Despite chemical transfection, for example, lipofection achieving low to moderate efficiencies for difficult to transfect cells such as stem cells and cardiomyocytes derived from induced pluripotent stem cells (iPSCs) (Giacalone et al., 2018; Tan et al., 2019; Roig-Merino et al., 2022), its affordability makes it a common choice in resource limited settings.

### 3.2 The enrichment of precise gene edited clones: methods of selection

Clonal selection is required to increase the frequency (accuracy and quantity) of obtaining precisely edited cells from a population consisting of edited and unedited cells, examples of the enrichment of gene edited cells (Figure 2B). This crucial process of improving the editing efficiency is performed either by supplementing cultures with antibiotics, sorting via fluorescence activated cell sorting (FACS) or magnetic bead separation, (Figure 2C). Co-expression of selection markers such as GFP or antibiotic resistant genes significantly enhances clonal selection. This can be accomplished by using plasmids with selection markers tagged via an IRES element or a self-cleaving 2A peptide (Box 1) to ensure the same cells expressing the genome engineering components express the selection marker, which is particularly useful in cells refractive to transfection. GFP expressing cells can be selected via FACs or microscopy, whilst antibiotic selection effectively eliminates cells that have not been transfected (Ibrahimi et al., 2009). The enrichment of individual clonal cell lines with the incorporated genetic modification is advantageous, as this will reduce the resources required to validate genetically edited clones, which generally involves the laborious process of screening over hundreds of clones per edit in the absence of an enrichment method (reviewed extensively by Mikkelsen and Bak, 2023).

Limitations to be considered when selecting an enrichment method include sorting efficacy, time, labour and cost (Ramachandran et al., 2021). Antibiotic selection (Figures 2B, C, right column) is considered a cost-effective strategy for screening iPSC edited cells without the requirement of expensive cell sorting instruments (Yadav and Thelma, 2022). FACS (Figures 2B, C, left column) implements automated single, live cell collection based on the expression of a fluorescent reporter system, which is costly and causes significant cell stress (Kim et al., 2014). Yet, it is this automation of cell sorting by FACS that would generally be favoured over manually selecting edited clones from a population in instances where a large number, for example, 384 clones are required to be screened (Caillaud et al., 2022). However, to bypass this technique in the absence of the skills and costly infrastructure, Li et al. performed limited dilutions to implement a multiplex prime editing strategy to allow for single cell clonal expansion and screening (Li et al., 2022).

A standard FACS instrument without reagents (primary or secondary antibodies) costs approximately $185 000 and the operation of such an instrument requires specialized training. The combined analysis, generating qualitative and quantitative data with the ability to enrich for cell populations by this powerful technique is advantageous, yet for the purpose of selection there are more cost-effective strategies available. Magnetic-activated genome edited cells sorting (MAGECS, Figures 2B, C, center column) is an efficacious cost-effective technique to enrich for edited cells engineered with a surface marker such as CD19. In this study, a SpCas9- CD19 co-expression plasmid was delivered by lipofection into HEK293T and iPSCs achieving a 4-fold and 3-fold improvement in reporter efficiency post MAGECS compared to cells pre-sorting as evaluated by immunofluorescence microscopy (Ramachandran et al., 2021). Unlike antibiotic selection and FACS, experimental optimization that traditionally require laborious ‘kill curves’ and specified instrument parameters for FACs, respectively, are not necessary for enrichment with MAGECS (Ramachandran et al., 2021). Multiplexing with several surface markers and the reusability would be a key consideration for future development of the MAGECS technology. With the sole purpose of enriching for edited clonal lines, antibiotic enrichment may be the most economical solution for the first-time user with limited research funding followed by MAGECS, which was found to cost twice in comparison.

Newer bioengineering technologies, which are derived from the CRISPR-Cas9 system have improved precise editing by creating large template knockins (1–100 kb) and reduce genotoxicity without invoking the endogenous cellular repair pathways arising from DSBs. However, reduced integration efficiencies obtained in iPSCs with the use of these technologies have been mitigated by enrichment methods imparted by the expression of antibiotic resistance genes or fluorescent protein encoding genes (Blanch-Asensio et al., 2022; Wang et al., 2022; Lampe et al., 2023; Yarnall et al., 2023). Blanch-Asensio and colleagues devised a multiparameter reporter (_˜_14 kb in length) and multiplexed genetic variants (a 50 kb DNA sequence for twelve variants associated with cardiac arrhythmic disorder) in iPSC lines using antibiotic enrichment to improve integration efficiencies (of the integrase landing pads) from 2.0% to *>* 99.4% post enrichment (Blanch-Asensio et al., 2022; 2023). This integration of large payloads can be achieved by site-specific integrases (Box 1), applied to mammalian cells a decade prior to CRISPR technologies (Groth et al., 2000). Compared to the conventional means of gene insertion that is reliant on HDR after DSBs, the efficiency of integration decreases with an increase in repair template size. These integrases function independent of the endogenous repair pathways and DSBs to integrate large payloads (Byrne et al., 2015). In this study, recombination is mediated between attachment, attP sites, which are pre-installed using CRISPR-Cas9 at the target locus and the attB sites residing in DNA cargo cassette (Thorpe and Smith, 1998; Blanch-Asensio et al., 2022). In conjunction with this selection, iPSCs were clonally isolated using FACS instrumentation using co-expression of mCherry and zeocin in the reporter iPSC lines generated. Near scarless genomic integration was achieved bar the incorporation of loxP sites, which is required for Cre-Lox (Box 1) mediated recombination to excise the genes expressing the selection markers post enrichment (Sternberg and Hamilton, 1981; Davis et al., 2008; Brandão et al., 2022). The drawback of the lengthy molecular cloning process orchestrated to generate the plasmids in this study, is however compensated for by an improvement in integration efficiency achieved, which is a challenge when using recombinase technologies (Fichter et al., 2023). Another study demonstrated the enrichment of cells that have undergone HDR by developing a split puromycin reporter system engineered with a cloning site lodged between a prematurely terminated 5′ puromycin coding sequence and the full-length coding sequence (Flemr and Bühler, 2015). Similar to this split puromycin system, precise integration of the repair template corrected the reading frame of the selection marker to promote zeocin resistance gene expression in correctly integrated iPSCs (Blanch-Asensio et al., 2022). These studies emphasize the benefit of improving genome engineering efficiency particularly in a resource constrained setting to reduce both expense and time required to obtain clonal edited lines.

## 4 Cost constrained profiling of a genome engineered cell population

A myriad of simplified techniques can be applied to evaluate the effects of CRISPR-Cas9 mutagenesis at targeted loci. In aligning with the focus of this review, indel detection methods, which are both cost constrained and easily implementable are provided here. In depth insights that surveys the available methodologies for precise indel detection have been reviewed elsewhere (Sentmanat et al., 2018; Bennett et al., 2020). Semiquantitative validation by PCR allows for the detection of large deletions generated by multiple gRNA excision, mismatch heteroduplex cleavage (Mashal et al., 1995; Oleykowski et al., 1998) and restriction fragment length polymorphisms is used to assess for the absence or presence of restriction enzyme site incorporation subsequent to precise repair (Botstein et al., 1980). These methods are generally coupled with sequencing technologies varying from less costly, Sanger sequencing to more costly, NGS. However, only a select number of research labs have the required expertise and resources to accomplish the more costly yet sensitive, deep coverage sequencing used for identifying rare editing events. Table 2 summarizes these cost-effective strategies, outlining the purpose, sample input required, ease of use, cost, limitations, and the process to be followed for clone profiling (if deemed necessary).

**TABLE 2:** Cost constrained, implementable functional assays for evaluation of a genome engineered population.

Assay	Genetic modification	Sample input	Time and throughput ease, speed and suitability of a technique for profiling	Simple to more laborious	Cost	Reliability and limitation	Precision	Next step required
Multiple excision	Deletion products for KO generation	PCR amplified samples assessed by gel electrophoresis	Medium throughput and rapid method	Simple	Low	Reliable for multiple gRNA excision to pre-screen deletion mutants Monoallelic deletions are indistinguishable	All products amplified by PCR	Bacterial cloning and Sanger sequencing of dominant mutations to identify monoallelic and biallelic modifications
Cel I	On-target editing and estimates indel efficiencies	gDNA (1x confluent 96-well) PCR purified, Denatured + reannealed	Low throughput and labor-intensive 4 h (Cleaving heteroduplex and separation by PAGE)	More laborious	Low, if crude Cel I from celery extract is prepared	Limit of detection of 3% Requires spike treatment to ensure efficient cleaving of heteroduplexes using crude Cel I extract	-Semi-quantitative -Identifies transition and transversion mutations -Cannot distinguish homozygous WT or homozygous mutant -Cannot resolve monoallelic and biallelic modifications -False positives arising from endogenous SNPs	As above
T7EI	On-target editing and estimates indel efficiencies	gDNA (1x confluent 96-well) PCR purified, Denatured + reannealed	Low throughput and labor-intensive 4 h (Cleaving heteroduplex and separation by PAGE)	More laborious	Medium	Detection limit at 1%	As above Specifically identifies indels mutations	As above
RFLP RFLP cont	On-target editing and estimated HDR efficiency OR On-target, loss of RE site	gDNA (1x confluent 96-well) PCR purified	Low throughput and labor-intensive 4 h (Cleaving and separation by gel electrophoresis)	More laborious	Medium	Higher concentrations of restriction enzyme required to induce sufficient cleavage thereby avoiding false negative results Restriction enzyme site within repair template to be installed within 10 bp of DSB to be effective or Indel must destroy RE site	-Presence or absence precisely defined -Recognizes the WT from mutant and monoallelic mutants	As above
Sanger sequencing	On-target from 1bp indels	As above	High-throughput and labor-intensive. Ensure forward or reverse primers is situated >50 bp from DSB	More laborious	High, all (unedited/edit) sequenced increasing cost	-Access to sequencing facility -Requires a homogenous cell population that have been expanded clonally to distinguish monoallelic events -Potentially not sensitive to detect rare/less frequent mutations	-Limit of detection for large insertions (>1 kb) or small deletions present in low abundance are undetected -First 50 bp of read can be unresolved, biasing trace peak analysis -Bacterial cloning required for allelic analysis in a mixed population	Data analysis requires experience. TOPO cloning of mixed population followed by additional sequencing

(Mashal et al., 1995; Qiu et al., 2004; Huang et al., 2012; Yang et al., 2013b; Brinkman et al., 2014; Paquet et al., 2016; Brinkman et al., 2018b).

### 4.1 Cost constrained pre-screening of the targeted locus by heteroduplex analysis

**FIGURE 3:**
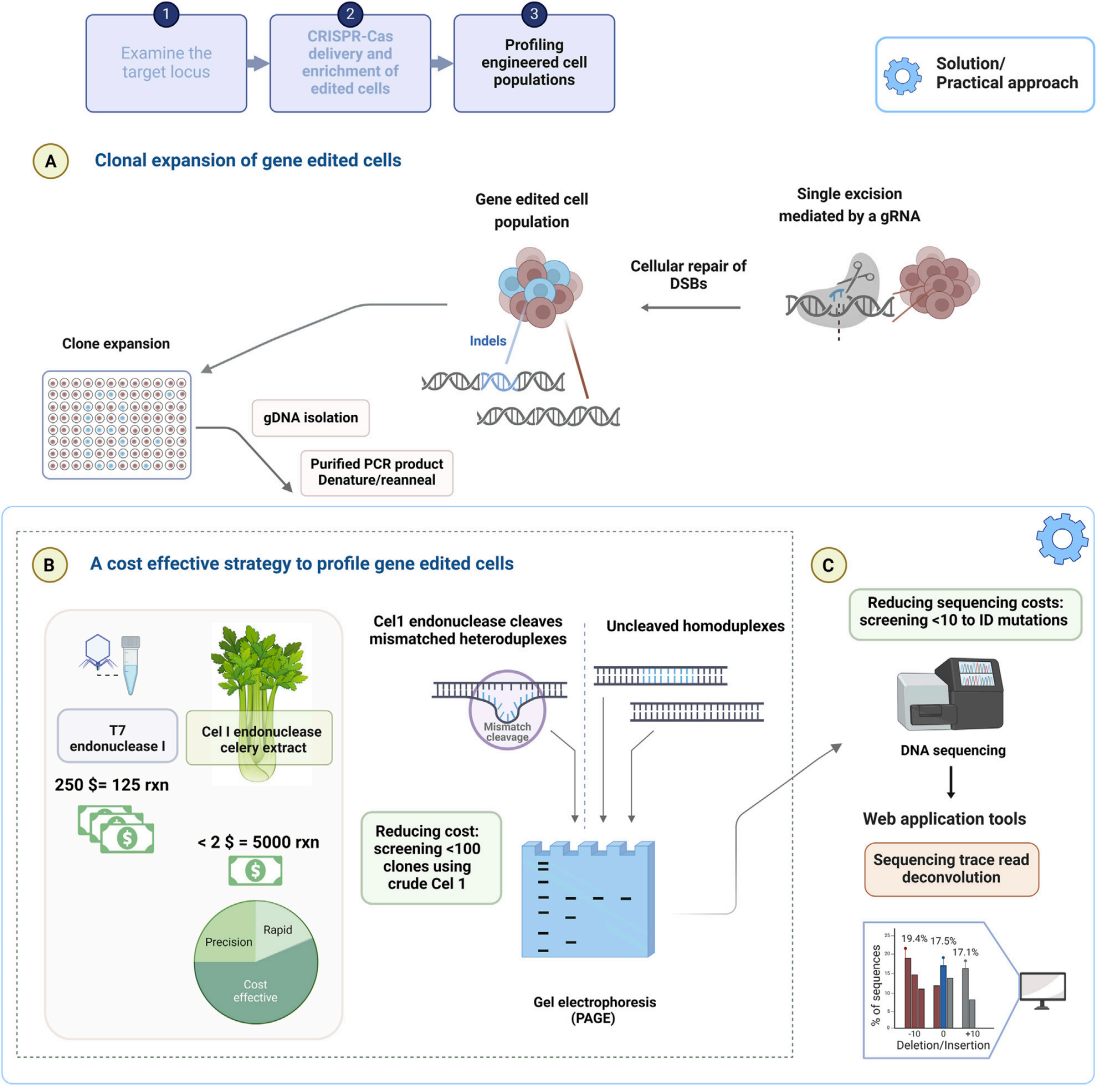
The experimental outline of a genome engineering experiment using the CRISPR-Cas9 system. **(A)** CRISPR-Cas9 based mutagenesis of a global edited population are clonally expanded to be **(B)** profiled using the cost effective, crude Cel I endonuclease extract for indel detection. **(C)** Prescreened clones are sequenced to confirm the identity of precise mutations attained, which can be supported by web application tools providing a % of sequences that contain either deletion, insertion compared to the reference sequence. Created with BioRender.com.

Figure 3 depicts the experimental workflow from transfection to clonal expansion (Figure 3A), the profiling of clones (Figure 3B) and end point identification by sequencing (Figure 3C). The seminal publications introducing CRISPR-Cas9 genome engineering probed for minor indels in the targeted loci resulting from the error prone repair of DSBs made use of the Surveyor nuclease assay, whereby Cel I endonuclease cleaves mismatched DNA (Cong et al., 2013; Jinek et al., 2013; Hsu, et al., 2013b). Similarly, high performance gRNAs have been traditionally selected by using these mismatch cleavage assays (Vouillot et al., 2015). The mismatch cleavage assays (Figure 3B) are reliant on the cleavage of heteroduplexed PCR products formed by the rehybridization of WT-mutant sequences and un-cleaved homoduplex PCR products, which are produced by the rehybridization of WT-WT or mutant-mutant sequences) visualised with gel electrophoresis. The assay is subject to bias, as densitometry of band intensities (using gel electrophoresis) is estimated by imaging software to provide editing frequency. T7EI demonstrates higher sensitivity in the detection of deletion products, whereas Cel I outperforms in single nucleotide change detection (Mashal et al., 1995; Qiu et al., 2004; Vouillot et al., 2015).

Owing to the ease and cost effectiveness (<$5 for 1 kg of celery), methods (homogenized extract prepared with a juice extractor) of isolating Cel I from kilogram quantities of celery, crude Cel I extracts are effective for distinguishing unedited and edited clones that are isolated from a genome engineered population (Oleykowski et al., 1998; Yang et al., 2000). T7EI is recombinantly produced using the heterologous expression host, *Escherichia coli* with several methods aimed to improve yields and negate the resultant toxicity (of expression system) and is therefore, considered costly at $250 (1.25 mL of which 1 µL is generally used to digest <100 ng DNA) for high throughput screening of clones (Hadden et al., 2001). This pre-screening approach allows a researcher to screen a multitude of clones in a cost-effective manner. A cost comparison of Cel I extract produced in the lab and commercially purchased T7EI is shown in Figure 2B. For example, ten clones are sequenced from an initial fifty clones, thereby justifying the need for fewer Sanger sequencing reactions and hence lower cost to identify the precise nucleotide sequence and indel size (Figure 2C).

### 4.2 Cost-constrained assessment of pre-screened edited clones by sequencing and computational tools

Sanger sequencing is the gold standard for profiling a gene edited clone (Figure 2C) yet remains costly owing to the labour intensity of sample preparation, requirement for reagents, sequencing equipment and is therefore outsourced to core facilities. This method achieves <1 kb fragment read lengths at a cost of approximately $10 sequencing in forward and reverse directions and $15 for primer pair synthesis that is sourced from local sequencing facilities based in South Africa. In comparison to international genomic services rates this costs 10x more ($1 per kb) (*The Race for the $1,000 Genome |* Psomagen, 2015) as an example in South Africa. An ambitious research budget and resources would be required for deep sequencing technologies that are aimed at routine indel detection for institutions based in LMICs. Furthermore, Kingsmore and colleagues estimated the cost of NGS for an LMIC to be $23 per individual genome over a decade ago and this would decline by 10-fold every 18 months (Kingsmore et al., 2012). However, this reduction in Sanger sequencing let alone NGS sequencing costs has not been realised for LMIC countries.

Sequencing data representative of an edited population can be evaluated using several freely accessible, web applications (TIDE, CRISP-ID, TIDER, DECODR and ICE) that deconvolute Sanger trace reads to establish an alignment window between the isogenic control, reference unedited sample and the mutated edited sample (Figure 2C). The reference sequence and the mutated edited sequences are obtained from expanded clones from the user’s global edited population to provide a reliable assessment of editing outcomes. These online tools require gRNA sequence input and report the identity of indels and frequencies in genome engineered populations with some reporting on precise editing generated by HDR (Brinkman et al., 2014; Dehairs et al., 2016; Brinkman et al., 2018b; Bloh et al., 2021; Conant et al., 2022). These web applications provide rapid and efficient insight into the mutation spectrum of a cell population at no cost and can be beneficial to implement for all investigators performing gene editing research.

## 5 Discussion

The insights we have attempted to convey here are aimed towards first-time users with limited available resources and research funding. This is contextualized by the commonly followed workflow to undertake genome engineering research, which involves scrutinizing the target loci and implementing essential controls (transfection control, gRNA controls, etc.). We have attempted to outline how to implement CRISPR technology in a limited R&D funding environment, that in our experience, might be helpful to those with capability but limited resources.

The reasons for low editing efficiency could potentially be the result of an occluded chromatin landscape, or the crucial nature of the targeted gene, in cell survival (Gluecksohn-Waelsch, 1963; Hart et al., 2014; Shalem et al., 2014; Wang et al., 2014). Databases such as the UCSC genome browser, http://genome.ucsc.edu (Kent et al., 2002) provide valuable information on the gene transcript by considering gene expression levels in the assigned cell type (e.g., genotype-tissue expression, GTEx (Lonsdale et al., 2013)). In addition, low gene expression levels as a consequence of an occluded chromatin could also be conclusively validated by qPCR. Similarly, the change in protein production levels measured by Western blot may provide a functional readout of the genetic modification engineered, for example, a gene knockout. The current experimental workflow remains a constant, published about a decade ago (Ran, et al., 2013b). These can be structured into 3 categories, firstly free-to-use, *in silico* evaluation for the design of CRISPR components (Figure 1), secondly the optimization of transfection and enrichment of edited clones (Figure 2) and lastly the validation of the edit (Figure 3). The first category involves meticulous, preliminary analysis of the target loci, followed by selecting the delivery format, gRNA design including the repair template for HDR dependent gene/SNP correction, and gene editing assay development, which requires primer design, restriction enzyme selection for screening of edited clones. The second category necessitates the optimization of transfection and selection by transient expression of an antibiotic resistance gene. Transfection efficiency dictates whether the CRISPR components are delivered into the cell, and this is visualized by a fluorescent tag subsequently followed by the selection of high-performance gRNAs. An equilibrium between cytotoxicity caused by the transfection of CRISPR components and antibiotic treatment is then achieved to obtain the desired genome engineered cell line. The last category is the evaluation of the gene editing outcome by mismatch cleavage assays and sequencing of the targeted locus and providing a definitive readout (validation) of the bioengineered phenotype, using proteomics, metabolomics.

The recommendations provided are for performing CRISPR-Cas9 genome engineering *in vitro*. Established cell lines, particularly iPSCs may acquire genetic changes over prolonged cell culture periods, which may, for example, confer altered growth rates that are repeatedly caused by mutations in the *TP53* gene (Merkle et al., 2017). In addition, the p53-mediated DNA damage response mediated by CRISPR-Cas9 DSBs triggers tumorigenic potential to modified lines, and numerous researchers recommend the evaluation of functional p53 prior and subsequent to gene editing (Ihry et al., 2018). A p53 reporter assay was developed to assure the integrity of the p53 pathway in the established iPSC reference lines to be utilized for future research endeavors (Pantazis et al., 2022). Aside from the off-target effects, another concern is the deleterious on-target editing events that are frequently overlooked by conventional locus sequencing that negatively impact the study reliability (Weisheit et al., 2020). “DSB-free” renditions of CRISPR-Cas9 are promising to negate the challenges observed, however recent studies have uncovered genotoxic effects, observing DSBs with deletions and translocations mediated by base editing and prime editing although at lower frequencies compared to Cas9 mediated DSBs (Fiumara et al., 2023).

Whilst the field of genome engineering technology continues to expand exponentially, the underrepresentation of diverse genomic data from Africa precludes such a region from realizing the true potential CRISPR genome engineering holds. A serious flaw resides in the gRNA design platforms, which make use of reference genomes that do not represent these diverse genomes (Misek et al., 2024). The gap in health disparities is being bridged by reference consortiums such as the human pangenome and others that aim to include ethnically diverse populations to identify unreported variants (Auton et al., 2015; Fan et al., 2023; Liao et al., 2023). Inclusion of diverse genomic data from under-represented regions will ensure that all off-target effects are taken into account, thus mitigating undesired, deleterious outcomes and providing equitable gene editing efficacy across all populations (Lessard et al., 2017; Canver et al., 2018; Misek et al., 2024). Other barriers hindering the widespread use and implementation of genome engineering include the lack of infrastructure, skilled researchers, theoretical and practical initiatives (that keep abreast with global research), adequate funding and sustainable global and national collaborations. As researchers in this field, with limited funding and resources we will continue in our endeavours to democratize the utility of genome engineering technology for LMICs, thereby providing solutions aimed at improving the livelihood of the Global South. It is an encouraging assumption that as sequencing costs for high income countries were once considered expensive but through the years has become affordable, gene editing reagents will most likely become cost effective for LMIC and readily available, thereby remedying the dearth in its utility within African countries and others with limiting resources. In the interim–we hope this review will assist the first-time user to effectively navigate the hurdles imposed by unjustifiable cost barriers to achieve affordable and efficient gene editing.

## Abbreviations

ChIP-seq, chromatin immunoprecipitation-sequencing; Cas, CRISPR-associated protein; crRNA, CRISPR RNAs; DSB, double stranded break; FACS, fluorescence activated cell sorting; HDR, homology directed repair; indels, insertions and deletions; IRES, internal ribosome entry site; LMICs, low- and middle-income countries; MMEJ, microhomology mediated end joining; NHEJ, non-homologous end joining; NMD, nonsense mediated decay; PAMs, protospacer-adjacent motifs; PTCs, premature termination codons; RNP, ribonucleoprotein; (s)gRNA, (single) guide RNA; T7EI, T7 endonuclease I; tracrRNA, trans-activating CRISPR RNA.
